# Strangulated Ileus Caused by Clips Used for Closure of an Appendiceal Endoscopic Submucosal Dissection Perforation: A Case Report

**DOI:** 10.1002/deo2.70383

**Published:** 2026-07-10

**Authors:** Miuzen Kanamori, Yohei Minato, Susumu Banjoya, Tomoya Kimura, Koichi Furuta, Nao Takeuchi, Rikimaru Sawada, Shouichi Satou, Yuhki Sakuraoka, Ken Ohata

**Affiliations:** ^1^ Department of Gastrointestinal Endoscopy NTT Medical Center Tokyo Tokyo Japan; ^2^ Department of Surgery NTT Medical Center Tokyo Tokyo Japan; ^3^ Utsunomiya Daiichi Hospital Utsunomiya Japan

**Keywords:** clips, endoscopic submucosal dissection, ileus, obstruction, perforation

## Abstract

Perforation developing during endoscopic submucosal dissection (ESD) for colorectal lesions can necessitate halting of the procedure and performing emergency surgery. In some cases, conservative treatment of the perforation is possible using endoscopic clips or over‐the‐scope‐clips (OTSCs). In the case presented herein, while perforation closure was successfully achieved with conventional through‐the‐scope clips (hereafter referred to as “conventional clips”), the patient developed ileus as a complication, necessitating emergency surgery. The patient was a 69‐year‐old man who developed strangulated ileus caused by conventional clips placed for closure of a bowel perforation developing after ESD for a lesion in the cecum. We placed several conventional clips and OTSCs to close the perforation and confirmed the absence of any leak at a second‐look examination. However, 10 days later, the patient visited our emergency department complaining of abdominal pain. Abdominal contrast‐enhanced computed tomography revealed that a segment of the small intestine was drawn into the closing device, resulting in the development of strangulated ileus. We performed laparoscopic surgery and identified the conventional clips that had become caught in the small intestinal serosa/mesentery and led to the formation of a closed bowel loop and bowel obstruction. We released the loop without resection of the intestine. The damaged serosa and mesentery were sutured, and the patient was discharged on hospital day 8. Based on our experience, we suggest that the treating surgeon should bear in mind that even conventional clips used for perforation closure have the potential to grasp organs adjacent to the wall and cause obstruction and the development of symptoms after hospital discharge.

## 1 Introduction

Endoscopic submucosal dissection (ESD) for cecal lesions is associated with a relatively high risk of perforation [1], and over‐the‐scope‐clips (OTSCs) and conventional clips are often used for defect closure [2]. To our knowledge, this is the first reported case of bowel obstruction caused by conventional clips placed for closure of a cecal ESD‐related perforation.

## 2 Case Report

The patient was a 69‐year‐old man who was referred to our hospital for the treatment of a 0‐IIa lesion next to the appendiceal orifice measuring 20 mm in diameter. The patient had no history of prior abdominal surgery. His preoperative blood tests revealed no abnormalities. We found a 12‐mm reddish 0‐IIa lesion that edged slightly into the appendiceal orifice. The lesion was clearly demarcated and showed no surface irregularity, which was classified as a 2A lesion according to the Japan Narrow‐Band Imaging Expert Team classification, thus confirming it as an adenoma (Figure 1). We judged that because of its difficult location, endoscopic mucosal resection could result in piecemeal resection or incomplete removal, and therefore decided to perform ESD under general anesthesia. The lesion was excised with the Dual Knife (Olympus) in 65 min. The initial incision was started from the anal side of the lesion, with traction provided by the thread ring method [3] during the procedure. A small perforation was identified in the cecum. We initially attempted closure with conventional clips by grasping the muscular layer at the ulcer base; however, complete closure could not be achieved. Therefore, OTSCs were applied, followed by additional conventional clips for reinforcement. The closure required 95 min. We conducted a second‐look examination on day 3 after the ESD to confirm complete closure of the perforation—we found no leak, and all the OTSCs and most of the conventional clips were found to remain in place. The patient was discharged on day 6 after surgery in the absence of any complications at that point. Postoperative histopathological examination revealed that the lesion was a tubular adenoma, with negative surgical margins. On day 10 after the surgery, the patient began to experience epigastric pain and nausea; he visited our emergency department on day 11. Physical examination revealed mild tenderness in the abdomen, but neither rebound tenderness in the umbilical region nor hypoactive bowel sounds. Vital signs were stable. Hematological examination revealed the following: white blood cells, 10,300/µL; neutrophils, 83.3%; hemoglobin, 14.9 g/dL; platelet count, 35.6 × 10^4^/µL; blood urea nitrogen, 20.1 mg/dL; serum creatinine, 0.75 mg/dL; and serum C‐reactive protein, <0.03 mg/dL. Abdominal contrast‐enhanced computed tomography revealed that the conventional clips placed at the earlier surgery had pulled in the ileum, causing obstruction, and the ileal segment on the oral side was dilated with fluid accumulation, suggesting the possibility of strangulated ileus (Figure 2). We decided to perform emergency laparoscopic surgery. Intraoperatively, we found serous fluid in the pelvic cavity. The ESD conventional clips were found to be sticking out of the colonic wall and grabbing a part of the small intestinal serosa and the mesentery at a point 20 cm on the oral side, resulting in the formation of a closed loop (Figure 3a,b). The intestinal wall was congested, but there was no discoloration. We performed blunt dissection to release the loop and removed the conventional clips from the small intestine under direct vision by pulling the segment out from the abdominal cavity. The damaged serosa was reinforced by suturing with the muscle layer. No resection was performed, and the operative procedure was successful. He was discharged on day 8 after the surgery. A follow‐up colonoscopy performed 2 years later revealed no conventional clips other than the OTSCs.

**FIGURE 1:**
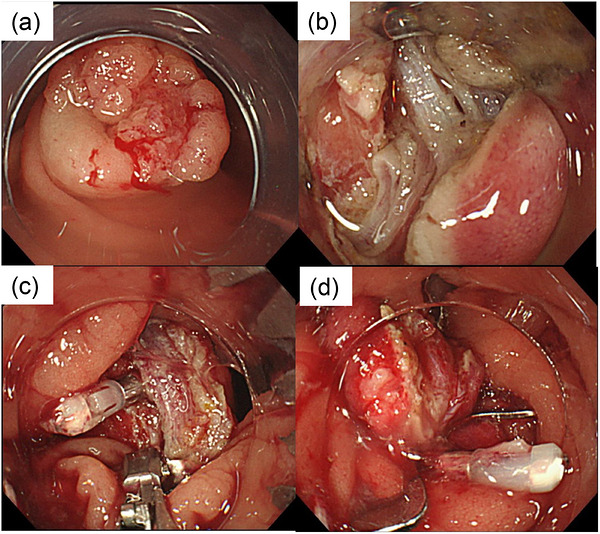
(a) Endoscopic findings at the previous clinic. 12‐mm 0‐IIa lesion in the cecum. (b) The cecum after endoscopic submucosal dissection (ESD). (c) The perforation after ESD. (d) The lesion was closed with conventional clips and Over‐the‐Scope‐Clips (OTSC).

**FIGURE 2:**
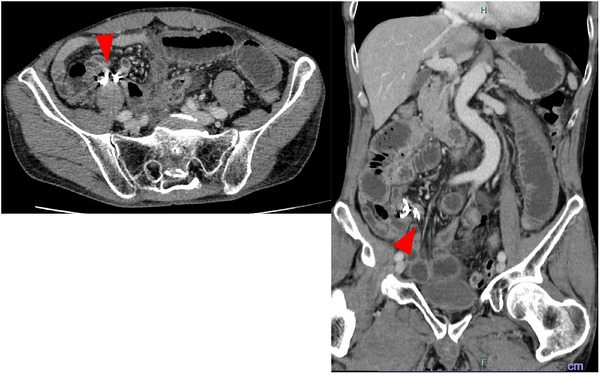
Contrast‐enhanced abdominal computed tomography at our hospital. The small intestine is dilated and edematous, but no free air is noted. The red arrowhead indicates the perforation‐closing device used after endoscopic submucosal dissection (ESD).

**FIGURE 3:**
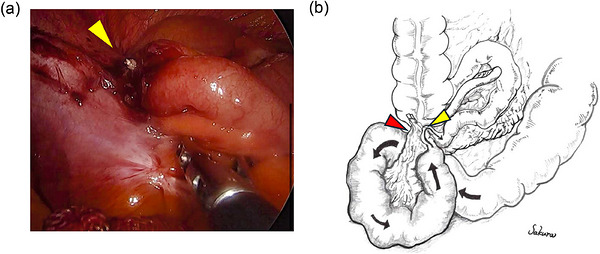
(a) Findings during surgery. The yellow arrowheads show a conventional clip sticking out from the colon. The conventional clip was found to be grasping the small intestine, resulting in the formation of a closed loop. (b) The figure shows a magnified picture of the loop. The yellow arrowhead indicates the anal side of the closed loop. The red arrowhead indicates the oral side of the closed loop, located approximately 20 cm proximal to the adhesion site. Clip hooking caused intestinal tethering followed by endoscopic submucosal dissection (ESD)‐related fibrosis and adhesion formation, resulting in kinking, angulation, progressive small bowel stenosis, and closed‐loop obstruction.

## 3 Discussion

ESD for lesions of the cecum is associated with a relatively high risk of perforation [1], often necessitating closure using conventional clips [4]. The complications related to the use of conventional clips include hemorrhage, tissue damage, embedded conventional clips in tissues/plaques, and bowel perforation [5]. However, to our knowledge, bowel obstruction caused by conventional clips used for closure of an ESD‐related perforation has not been reported. Although adjacent organ involvement has been reported after OTSC placement [6], ileus caused by conventional clips grasping the adjacent small intestine or mesentery appears to be extremely rare. It is important to pay careful attention to anatomical issues to prevent further complications from developing when closing a perforation after ESD. Through the experience of this case of obstruction caused by the use of a closing device, we consider that since endoscopists grasp enough tissue tightly while trying to cover a perforation completely, perforation closure could cause obstruction regardless of the size of the closing device.

In regard to the technique for perforation closure, the conventional clips grasped the mesentery, achieving perforation closure similar to an omental patch but likely contributing to closed‐loop formation. Omental patch–assisted closure, although effective for selected difficult perforations [7], may increase the risk of unintended tissue entrapment depending on the perforation site. Therefore, careful approximation of the edges of the defect, and where appropriate, use of a twin grasper should be considered [8].

With respect to the time to the development of ileus in our patient, it was another 10 days until the patient developed some symptoms. In our present case, conventional clips used for perforation closure got hooked to the cecum, and the following fibrosis led to adhesion of the small intestine to the cecum, resulting in kinking and angulated small bowel stenosis. Thus, the closed‐loop obstruction appeared to develop through a two‐step process. Initially, both the oral and anal sides of the loop were tethered to the cecum by the conventional clips, which we assume stuck out from the point of perforation closure, although the clips themselves did not initially cause critical narrowing of the small bowel lumen. Then, traction of the small bowel by the clips led to adhesion formation, resulting in progressive stenosis and eventual development of a closed‐loop small bowel obstruction. During the healing process, it takes approximately one to two weeks for the migration of fibroblasts and formation of a fibrin band [9]. Therefore, formation of a closed loop attributable to adhesion between the injured serosal surfaces and the adjacent intestinal mesentery could take one to two weeks. Based on this perspective, the reason why the obstruction was not detected in our patient while he was still hospitalized after the first surgery was that a length of time was needed for the adhesions to develop. From an anatomical standpoint, the cecum is adjacent to the mobile small bowel and mesentery; therefore, perforation closure in this region may cause unintended tissue entrapment or fixation, not only directly by the conventional clips getting hooked to adjacent tissues, but also indirectly through the occurrence of extensive fibrosis [10].

This case is significant as it presents the first instance of strangulated ileus developing as a complication of endoscopic conventional clip placement to close a perforation after ESD. It can be considered a cautionary case highlighting the risk of conventional clips placed for complete closure of an ESD perforation, grasping and damaging adjacent organs. Endoscopists should also be aware that the obstruction takes time to develop and that patients may therefore only show symptoms after discharge. Therefore, careful follow‐up for a few weeks should be undertaken in cases undergoing perforation closure by clip placement after ESD.

## Author Contributions

**Miuzen Kanamori** wrote the article; **Miuzen Kanamori** and **Ken Ohata** conceived and designed the study; **Yohei Minato**, **Shouichi Satou**, **Nao Takeuchi**, **Koichi Furuta**, **Susumu Banjoya**, and **Rikimaru Sawada** provided patient management; **Yuhki Sakuraoka** provided visualization; **Ken Ohata** provided the final approval of the article.

## Funding

The authors have nothing to report.

## Ethics Statement

All procedures followed have been performed in accordance with the ethical standards laid down in the Declaration of Helsinki and its later amendments.

## Conflicts of Interest

The authors declare no conflicts of interest.
